# Cecal Volvulus as a Rare Complication of Osimertinib Dosed at 160 mg in Patients With *EGFR*-Mutant Non-small Cell Lung Cancer

**DOI:** 10.3389/fonc.2020.00510

**Published:** 2020-04-15

**Authors:** Tejas Patil, Jose M. Pacheco, Anastasios Dimou, William T. Purcell, Candice Rossi, Paul A. Bunn, Robert C. Doebele, D. Ross Camidge, Lisa Ferrigno

**Affiliations:** ^1^Division of Medical Oncology, University of Colorado School of Medicine, Aurora, CO, United States; ^2^Division of Medical Oncology, Mayo Clinic, Aurora, CO, United States; ^3^Department of Surgery, University of Colorado School of Medicine, Aurora, CO, United States

**Keywords:** EGFR, NSCLC, tyrosine kinase inhibitor, osimertinib, volvulus

## Abstract

**Background:** Osimertinib is a 3rd-generation tyrosine kinase inhibitor (TKI) that blocks the epidermal growth factor receptor (EGFR) in non-small lung cancer (NSCLC) and has dramatically improved outcomes for patients with EGFR mutations. While gastrointestinal complications such as diarrhea have been reported with EGFR inhibitors (due to off-target interactions with EGFR receptors within the gut lining), cecal volvulus is an extremely rare complication in advanced malignancy. To date, there are no reported cases associating cecal volvulus with any EGFR TKIs.

**Case Presentation:** In this case series, we present three cases of cecal volvulus among patients with EGFR-positive NSCLC patients treated with osimertinib dosed at double the standard 80 mg dose (160 mg daily). No patient was receiving concurrent chemotherapy or bevacizumab at the time of this described complication. In two cases where pathology was available for review, peritoneal carcinomatosis or intra-abdominal spread was not observed. In a retrospective evaluation of 101 patients treated with osimertinib in our institution, there was a statistically significant difference in the incidence of cecal volvulus among patients receiving osimertinib at 160 mg vs. patients receiving the 80 mg dose (27 vs. 0%; *p* < 0.001).

**Conclusions:** To our knowledge, these are the first cases to highlight a potentially important and serious gastrointestinal complication associated with the 160 mg dose of osimertinib.

## Introduction

The identification of the epidermal growth factor receptor (EGFR) in non-small lung cancer (NSCLC) has led to the development of tyrosine kinase inhibitors (TKIs) that inhibit these mutations with a high degree of specificity. Patients with activating *EGFR* mutations demonstrate improved outcomes with respect to objective response rate (ORR), progression free survival (PFS), and quality of life (QoL) due to the availability of selective and effective TKIs (1–6). Osimertinib is a third-generation irreversible TKI that overcomes T790M, the most common acquired resistance mutation to first generation EGFR inhibitors (1, 5, 7). Gastrointestinal complications such as diarrhea have been reported with EGFR inhibitors due to off-target interactions with EGFR receptors within the gut lining. While malignant bowel obstruction and bowel perforation are commonly seen in advanced cancer (especially in gastrointestinal and gynecological malignancies), volvulus is an extremely rare complication overall (8). To date, there are no reports of a relationship between EGFR inhibition and the development of volvulus. We report three cases of patients with EGFR mutant NSCLC who developed cecal volvulus after being treated with osimertinib at double the standard 80 mg dose (160 mg daily).

## Case Vignette 1

A 53 year old Caucasian female never smoker presented to her primary care physician with shoulder pain and was subsequently diagnosed with metastatic lung adenocarcinoma. Computed tomography (CT) of the chest, abdomen, and pelvis revealed a right middle lobe lung mass (5.2 × 4.5 cm), contralateral mediastinal lymphadenopathy, and numerous osseous metastases involving the vertebrae without epidural extension or cord compression. Brain magnetic resonance imaging (MRI) did not find metastatic disease at the time of diagnosis. Endobronchial biopsy of the right middle lobe lung mass was positive for poorly differentiated lung adenocarcinoma. Next-generation sequencing (NGS) from this sample revealed an *EGFR* L858R point mutation. She was started on erlotinib 150 mg PO daily with an excellent partial response to therapy. She did not receive chemotherapy or bevacizumab prior to starting erlotinib. Approximately 8 months after receiving erlotinib, she received intensity-modulated radiotherapy (5,000 cGy over 10 fractions) to three oligoprogressive lung lesions. After 10 months from radiotherapy, she progressed in the brain and left ulna. She received stereotactic radiotherapy (2,000 cGy over one fraction) to her left cerebellar vermis and switched to rociletinib, a third generation EGFR TKI, in the context of a clinical trial. She had a partial response to this therapy for 5 months before developing worsening headache, gait ataxia, and vision changes secondary to leptomeningeal progression. A CT abdomen and pelvis at time of progression on rociletinib found no peritoneal carcinomatosis or intraabdominal disease. She was switched to osimertinib dosed at 160 mg PO daily for increased intracranial penetrance. She had rapid resolution of her neurological symptoms. She remained on this therapy for 1 year, before being admitted to the hospital for acute right-sided lower quadrant abdominal pain associated with obstipation. Abdominal exam was notable for distension, rebound tenderness along the right upper quadrant, and involuntary guarding. Of note, her admission vitals were notable for bradycardia. A CT abdomen obtained in the emergency department demonstrated cecal interposition between the liver and the anterior peritoneum with mild dilatation of the cecum and swirling of the distal ileum about the ileocolic vasculature. She was taken emergently to the operating room where an exploratory laparotomy, right hemicolectomy, and end ileostomy were performed. There was no evidence of peritoneal carcinomatosis or malignant bowel obstruction by visual inspection of abdomen. Examination of the resected right colon demonstrated serosal adhesions, tortuous contour, and vascular congestion consistent with cecal volvulus. Osimertinib was discontinued after surgery. One month later, she was treated with IV carboplatin (AUC 6), pemetrexed 500 mg/m2, and pembrolizumab 200 mg with ongoing response.

## Case Vignette 2

A 66 year old Caucasian male never smoker developed a non-productive cough for 1 month that failed to respond to outpatient antibiotics, inhaled bronchodilators, and short courses of prednisone. His primary care physician obtained a CT chest that demonstrated a right upper lobe mass (5.2 × 4.7 cm) along with numerous satellite nodules in the right lower lung. He was admitted to an outside hospital where a CT-guided biopsy of the right upper lobe lung mass was performed. Biopsy from this specimen revealed nests of mucinous tumor cells with an immunophenotype negative for cytokeratin 20 (CK20), positive for cytokeratin 7 (CK7), and positive for thyroid transcription factor 1 (TTF-1) consistent with lung adenocarcinoma. Real-time polymerase chain reaction (RT-PCR) testing revealed an *EGFR* L858R point mutation. Staging positron emission tomography–computed tomography (PET/CT) identified a large fluorodeoxyglucose avid (FDG) lesion in the right upper lobe of lung along with subcarinal and ipsilateral mediastinal, paratracheal, and supraclavicular lymph nodes, but did not identify any extrathoracic disease. An MRI of the brain demonstrated a single right parietal lesion measuring 7 mm with surrounding vasogenic edema. This lesion was treated with stereotactic radiosurgery (2,000 cGy over one fraction). He received erlotinib 150 mg PO daily with an excellent partial response to therapy. He tolerated therapy well with Grade 1 diarrhea and acneiform rash as the principal adverse effects. After 2 years on therapy, he progressed in the liver and pancreas. Re-staging imaging with PET/CT did not demonstrate any peritoneal carcinomatosis. A CT-guided biopsy of the liver metastasis demonstrated an *EGFR T790M* mutation and he was switched to osimertinib 80 mg PO daily. His osimertinib dose was increased to 160 mg PO daily after 8 months for progressive CNS disease. He continued this therapy for ~10 months before being admitted to an outside hospital for acute right sided abdominal pain. On admission, he was found to have cecal volvulus requiring a hemicolectomy. Pathology from the resected specimen was not available for our review. Due to ongoing symptomatic CNS disease, he was restarted on osimertinib 160 mg PO daily 1 week after his hemicolectomy. Three weeks after restarting osimertinib at 160 mg, he developed progressive CNS disease. Assessment of circulating tumor DNA (ctDNA) identified *EGFR* L792V *in trans* with T790M. Based on this acquired resistance mutation, he was switched to afatinib with a partial response in the CNS.

## Case Vignette 3

**Figure 1:**
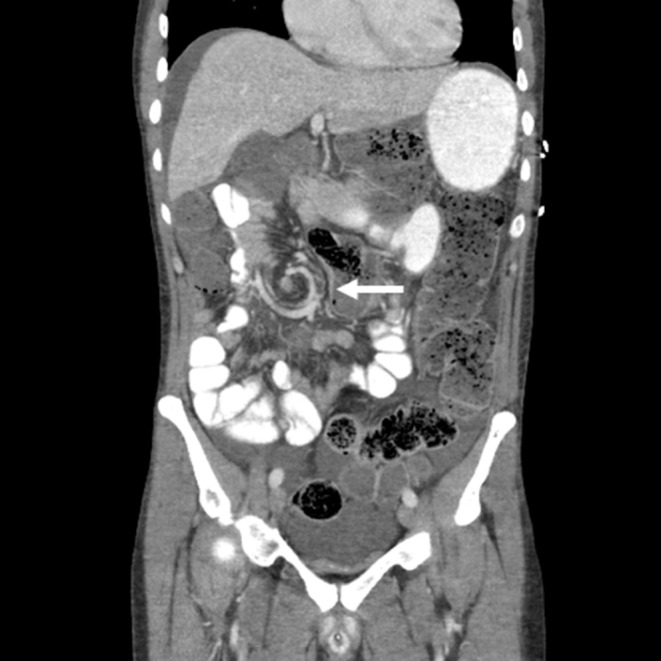
A CT scan of the abdomen demonstrates a “whirl sign” which is when the bowel rotates arounds its mesentery leading to whirls of the mesenteric vessels.

A 44 year old Caucasian male never smoker was diagnosed with metastatic lung adenocarcinoma after presenting to an emergency room with severe low back pain where he was found to have a lytic L2 lesion. CT chest imaging demonstrated a large pulmonary mass with irregular borders (3.2 × 3.2 cm) along the posterior basilar segment of the right lower lobe. Staging PET/CT demonstrated widespread metastatic disease including innumerable pulmonary nodules, ipsilateral pleural effusion, liver metastases, osseous metastases, and omental nodularity suspicious for peritoneal carcinomatosis. An MRI of the brain demonstrated two intracranial lesions involving the left occipital and left parietal lobe without vasogenic edema or midline shift. A core needle biopsy of the L2 vertebral lesion demonstrated malignant cells with immunohistochemistry negative for CK20, positive for CK7, and positive for TTF-1 consistent with lung adenocarcinoma. NGS of the decalcified bone sample revealed an *EGFR* Exon 19 deletion and a TP53 L114^*^ mutation. The patient received erlotinib 150 mg PO daily along with bevacizumab 15 mg/kg IV every 3 weeks with rapid resolution of his back pain. His first on-treatment PET/CT scan demonstrated marked response in all metastatic sites. He continued with this treatment regimen for 6 months before an on-treatment PET/CT scan demonstrated progression in T11, L5, S1 and the left acetabulum. A repeat bone biopsy from his L5 lesion demonstrated evidence of an *EGFR* T790M mutation and high-level *MET* amplification. *MET* copy number analysis was performed by fluorescence *in-situ* hybridization (FISH) testing and demonstrated a mean *MET*-per-cell of 13.87 and *MET*-to-centromeric enumeration probe for chromosome 7 (CEP 7) ratio of 5.04. Given the presence of both T790M and high *MET* amplification, he was switched to osimertinib 80 mg PO daily and crizotinib 250 mg PO BID (an FDA-approved ALK inhibitor with strong *MET* inhibition). He tolerated this combination well with improvement of his osseous metastases. He continued this combination for 8 months before developing left arm weakness and numbness. He was found to have new C3-C4 metastases with epidural extension and leptomeningeal disease on MRI of the brain and spine. Of note, the patient had no radiographic evidence of peritoneal carcinomatosis at time of leptomeningeal progression based on PET/CT. He was given a short course of dexamethasone 4 mg PO every 6 h and his osimertinib dose was increased to 160 mg PO daily. Palliative radiotherapy (3,000 cGy × 10 fractions) using three-dimensional conformal radiotherapy to C3-C4 was administered. He remained on the increased dose of osimertinib with crizotinib for 1 month before presenting to the emergency room with sudden onset of severe lower abdominal pain and distension. An abdominal CT scan on admission demonstrated swirling of the mesentery within the right central hemiabdomen at the level of the cecum/terminal ileum junction with significant gaseous distension of adjacent transverse colon (Figure 1). On evaluation in the emergency department, he was noted to be bradycardic (heart rate of 37) with a QTc of 429. He was emergently taken to the operating room where an exploratory laparotomy and right hemicolectomy with ileocolic anastomosis was performed; he was found to have partial malrotation, with the duodenum not crossing the midline, but fixed in the retroperitoneum and a mobile right colon. Pathological review of the resected right colon demonstrated edematous benign colonic mucosa with submucosal hemorrhage, vascular congestion, and vascular dilation. Despite initial concern for peritoneal carcinomatosis grossly, the mucosa was negative for dysplasia or invasive carcinoma. Multiple intra-abdominal nodes examined did not reveal any evidence of pulmonary adenocarcinoma. On post-operative day 1, he developed altered mental status and was found to have hemorrhagic conversion of a new brain metastasis. He was re-challenged with osimertinib 160 mg PO daily and crizotinib 250 mg PO BID 1 week after recovery from his surgical procedure given known leptomeningeal disease and the development of a new brain metastasis. Unfortunately, he went on to have a complicated post-operative course: almost 4 weeks after ileocolic resection with primary anastomosis and returning to work, he had anastomotic dehiscence for which he received an ileostomy and transverse colon mucus fistula in staged procedures. Two months after re-challenging with osimertinib 160 mg PO daily and crizotinib 250 m PO BID, he progressed in the pleura and multiple extrathoracic lymph nodes and was switched to IV carboplatin (AUC 6) and pemetrexed 500 mg/m2. He remained steadfast in his desire for stoma takedown after numerous discussions regarding the associated risks, which was performed 20 months after the stoma was performed; 4 weeks after takedown, he developed a fistula from the anastomosis. After his wound care was optimized, he was discharged with hospice care.

## Discussion

The etiology of cecal volvulus is likely related to late embryogenesis; the cecum rotates counterclockwise from the left side of the abdomen to the right lower quadrant. As this occurs, the mesentery of the right colon fixates to retroperitoneal structures (8). If the patient has incomplete fixation, there is risk of cecal volvulus formation. Based on the autopsy of 125 cadavers, 11.2% of right colons examined were freely mobile with complete common ileocolonic mesenterium, 25.6% were found to have cecum capable of “folding,” adding up to 36.8% of cadavers potentially at risk for cecal volvulus (8, 9). Known risk factors for cecal volvulus include chronic constipation, distal colon obstruction, high-fiber diets, ileus, prior colonoscopy, and late pregnancy (8). While malignant large bowel obstruction is a common complication of advanced gastrointestinal and ovarian malignancies, (10, 11) reports of malignancy-associated cecal volvulus are extremely rare and limited to case reports (12, 13). In an older case series of 37 patients with cecal volvulus seen during a 20 year period at surgical departments in Sweden, only two patients were found to have underlying gastrointestinal cancer at the time of surgical resetion (14).

Osimertinib received approval for use in NSCLC using an accelerated approval process. Common side effects of EGFR inhibitors include rash and diarrhea and are related to off-target inhibition of wild-type EGFR receptors distributed along the cutaneous and mucosal lining. Gastrointestinal complications with osimertinib dosed at 80 mg have been reported in two Phase 3 trials that resulted in osimertinib FDA approval (1, 5). Diarrhea was the most common Grade ≥3 gastrointestinal complication reported with 2 cases (1%) documented in the AURA3 trial and 6 cases (2%) documented in the FLAURA3 trial. Constipation, a known risk factor for volvulus, was only seen in 7 cases in the AURA3 trial, though neither trial reported any cases of Grade ≥3 constipation. In the phase 1 dose escalation study of osimertinib in patients with CNS metastases, there were increasing percentages of Grade 1–2 gastrointestinal toxicities observed at 200 mg (57%; 4/7), 300 mg (100%; 7/7), and 500 mg doses (100%; 3/3) (15). Only one patient had intolerable Grade 2 mucosal inflammation resulting in discontinuation of drug.

Numerous chemotherapeutic agents have been associated with bowel perforation and impaired wound healing, most notably anti-vascular endothelial growth factor (VEGF) inhibitors such as bevacizumab. The third patient in our series had previously been treated with bevacizumab, which could confound our interpretation. A retrospective series of 208 patients with NSCLC who received bevacizumab identified grade 3 diarrhea, febrile neutropenia, and stomatitis as risk factors for bevacizumab-associated perforation (16). Gastrointestinal perforation secondary to erlotinib, a first-generation EGFR inhibitor, is reported in two case reports (17, 18), but there have been no reports of bowel perforation associated with osimertinib.

To date, there has been no association with cecal volvulus and EGFR inhibition. In a review of 3540 osimertinib-treated cases from the FDA Adverse Events Reporting System (FAERS) database, only one case of gastric volvulus was described. A limitation of this database is that there are no data on dose and temporal association of drug delivery and adverse event. We performed a retrospective review of 101 patients treated with osimertinib in our institution. Group comparisons were performed using Fisher's exact test for categorical variables using *p* < 0.05 as cut-off for statistical significance. Cecal volvulus was more common among patients receiving osimertinib at the 160 mg dose (*n* = 11) vs. patients receiving the 80 mg dose (*n* = 90), a finding that was statistically significant (27 vs. 0%; *p* < 0.001).

There are two additional important observations in this series. The delayed wound healing described for the third patient in our series may be related to concurrent *MET* inhibition with crizotinib. The c-MET pathway has been identified as important for the process of wound healing (19). *MET* amplification is a described mechanism of resistance to EGFR inhibition, (20) and the addition of crizotinib, a *MET* inhibitor, is a strategy that is increasingly being utilized (21). While the concurrent use of crizotinib could influence the risk of volvulus, we have been unable to identify any cases of volvulus as a complication with crizotinib. Another important observation in this series was the presence of bradycardia at time of presentation among cases of volvulus, suggesting a potentially autonomic link with higher doses of osimertinib. In anatomically predisposed patients, higher doses of osimertinib may be contribute to a “second hit” resulting in the development of volvulus.

## Conclusion

To our knowledge, this is the first report associating cecal volvulus with the 160 mg dose of osimertinib. The correlation between three occurrences of a very rare event with all patients receiving the same drug at the same uncommon dosage seems unlikely to be a coincidence. The exact mechanism for this side effect is unclear and warrants further study. These cases highlight a potentially important surgical complication associated with the 160 mg dose of osimertinib.

## Data Availability Statement

The datasets generated for this study are available on request to the corresponding author.

## Ethics Statement

The studies involving human participants were reviewed and approved by Colorado Multiple Institutional Review Board (COMIRB). The patients/participants provided their written informed consent to participate in this study. Written informed consent was obtained from the individual(s) for the publication of any potentially identifiable images or data included in this article.

## Author Contributions

TP, DC, RD, and LF were involved with conception, design, and writing of the manuscript. All authors played a critical role in the appraisal of the final manuscript.

### Conflict of Interest

TP has received honoraria from PRIME Oncology and Genetech/Roche. JP has received honoraria or consulting fees Roche/Genentech, Takeda, AstraZeneca, GLG, Novartis, and Pfizer. PB received personal fees from AstraZeneca and Guardant Health. DC has received honoraria or consulting fees from Ariad, Takeda, Ignyta, and Roche/Genentech, and a sponsored clinical trial research agreement with Takeda. RD has consulting fees from Rain Therapeutics, Roche/Genentech, Takeda, AstraZeneca; a sponsored research agreement from Ignyta; licensing fees for patents from Abbott Molecular and Rain Therapeutics; licensing fees for biologic materials from Black Diamond, Pearl River, Ariad, Foundation Medicine, and Genentech; and stock ownership in Rain Therapeutics. The remaining authors declare that the research was conducted in the absence of any commercial or financial relationships that could be construed as a potential conflict of interest.
